# The development and preliminary psychometric properties of two positive psychology outcome measures for people with dementia: the PPOM and the EID-Q

**DOI:** 10.1186/s12877-017-0468-6

**Published:** 2017-03-21

**Authors:** Charlotte R. Stoner, Martin Orrell, Maria Long, Emese Csipke, Aimee Spector

**Affiliations:** 1 Research Department of Clinical, Educational and Health Psychology, University College London, London, UK; 2 Institute of Mental Health, University of Nottingham, Nottingham, UK; 3 Dementia Research Centre, North East London NHS Foundation Trust, Essex, UK; 4 Division of Psychology and Language Sciences, University College London, London, UK

**Keywords:** Dementia, Positive psychology, Independence, Hope, Resilience, Outcome measure, Psychometric

## Abstract

**Background:**

Positive psychology research in dementia care has largely been confined to the qualitative literature because of the lack of robust outcome measures. The aim of this study was to develop positive psychology outcome measures for people with dementia.

**Methods:**

Two measures were each developed in four stages. Firstly, literature reviews were conducted to identify and operationalise salient positive psychology themes in the qualitative literature and to examine existing measures of positive psychology. Secondly, themes were discussed within a qualitative study to add content validity for identified concepts (*n* = 17). Thirdly, draft measures were submitted to a panel of experts for feedback (*n* = 6). Finally, measures were used in a small-scale pilot study (*n* = 33) to establish psychometric properties.

**Results:**

Salient positive psychology themes were identified as hope, resilience, a sense of independence and social engagement. Existing measures of hope and resilience were adapted to form the Positive Psychology Outcome Measure (PPOM). Due to the inter-relatedness of independence and engagement for people with dementia, 28 items were developed for a new scale of Engagement and Independence in Dementia Questionnaire (EID-Q) following extensive qualitative work. Both measures demonstrated acceptable internal consistency (α = .849 and α = .907 respectively) and convergent validity.

**Conclusions:**

Two new positive psychology outcome measures were developed using a robust four-stage procedure. Preliminary psychometric data was adequate and the measures were easy to use, and acceptable for people with dementia.

**Electronic supplementary material:**

The online version of this article (doi:10.1186/s12877-017-0468-6) contains supplementary material, which is available to authorized users.

## Background

Outcome measures utilised within dementia research have generally been constructed using a loss/deficit approach, in which measures have been designed to examine co-morbid conditions such as anxiety [1] and depression [2] or behavioural and psychological symptoms of dementia (BPSD) [3]. Quality of life is often used as a primary outcome within research but this is not without issue. Often the overriding aim of such research is to reduce BPSD and it is inferred that this reduction should theoretically lead to an increase in quality of life [4]. However, this perspective has limitations as it does not include the perspective of the person with dementia.

Positive psychology may contribute to the concept of wellbeing for this population. Positive psychology refers to the study of positive emotions that enable individuals, communities and organisations to thrive [5], and advocates the use of quantitative methods to examine such positive concepts. Examples of positive concepts include humour, resilience, growth and spirituality. However, currently this approach is in its infancy and a lack of gold standard outcome measures means that positive attributes, experiences and outcomes are under researched for people with dementia [6] and the reporting of positive experiences of people with dementia is limited to the qualitative literature.

To examine the positive psychology concepts and their relationship to wellbeing, valid and reliable outcome measures are needed. The aim of this study was to develop such measures to be used in further research and clinical practice. It is hoped this will lead to a more asset based approach to the study of dementia and enable people to further live well with their diagnosis.

## Method

In order to develop these measures, we undertook a four stage process. Stage one consisted of a literature review in which evidence of positive psychology concepts from the qualitative dementia literature was sourced and examined. The most salient themes from these searches was developed into a topic guide for stage two. Stage two consisted of a qualitative study, in which positive psychology themes were explored with people with dementia and their carers to generate items for measures. Stage three consisted of expert feedback in which xdraft items within measures were examined for clarity, difficulty and content validity. Finally these measures were piloted with a small scale sample of people with dementia. These processes are discussed in more detail below.

### Stage 1: literature review

Qualitative literature published between 1998 and 2015 that investigated positive psychology concepts for people with dementia was examined. Search terms were: goal, self-efficacy, hope, resilience, coping, wisdom, growth, sense of coherence, control, autonomy, pleasure, self-realisation, sense of agency, gratitude, happiness, optimism, transcendence, positive, dignity, social participation, social inclusion, self-concept, humour, creativity, flow, spirituality, love, compassion, benefit finding, community integration, opportunity, social adjustment, mindfulness, acceptance, successful aging AND dementia, lewy body, vascular, Alzheimer, cognitive impairment, old, elder. Truncations of search terms were used where appropriate. For the purpose of this section, the Seligman definition of positive psychology [7] was used to screen results. This search enabled the authors to identify salient concepts in order to produce a topic guide for the focus group stage of development. Concurrently, searches were conducted to identify existing, robust outcome measures that could be adapted for people with dementia. Search terms above were used again but search terms denoting dementia were changed for the search terms: measure, instrument, questionnaire, quiz, test.

### Stage 2: focus groups

#### Design

A cross-sectional sectional qualitative design was used in which participants explored the meaning and implications of independence, social engagement, hope and resilience in dementia. Qualitative studies are recommended when designing outcome measures to ensure an adequate level of content validity [8]. A topic guide and semi-structured interview format were used to facilitate discussion and elicit in-depth views on subjective experience of this construct.

#### Participants

Ethical approval was obtained and participants were recruited through one private organisation (a care home in Leeds) and an existing Cognitive Stimulation Therapy (CST) group within a London NHS trust between September and October 2015. The CST group was identified as an appropriate source of recruitment for the focus group, as it was an established group consisting of people with mild dementia. People with dementia and carers were interviewed separately either individually or within a focus group, depending on preference and availability. 17 participants were recruited, all of whom met the inclusion criteria as detailed below:

#### Inclusion criteria

- People with a diagnosis of dementia according to the DSM-IV-TR criteria (American Psychiatric Association, 2000).
- Capacity to provide informed consent
- Able to communicate in English

OR

- People who identified themselves as an informal carer to a person with dementia.
- Capacity to provide informed consent
- Able to communicate in English

#### Data collection

Interviews followed a semi-structured format that allowed interviewers to ask spontaneous questions that addressed individual circumstances. This ensured sensitivity to participants’ self-expression with regard to constructing his or her own accounts. Sessions were audio-recorded and discussion was facilitated around individual meanings and expectations of independence, social engagement, hope and resilience within dementia, the barriers and facilitators to these concepts and potentially related constructs including reciprocity. This enabled items to be developed that accounted for the individual differences of these concepts according to different participants.

People with dementia participated in two focus groups. However, due to the logistical difficulty in gathering members of carers in one space, individual interviews (some by telephone) were conducted with these participants. Examples of questions posed to both people with dementia and carers included: “Tell me about your (your relatives/friends) experience of independence?”, “How has independence changed for you (your relative/friend/person you care for) since you began having memory problems?”, “How have social relationships changed for you (the person you care for)?” and “How does having people around you benefit or hinder you (the person you care for)?” Focus groups and interviews lasted between 16 min and 35 min and were largely dependent on the carer’s availability for individual interviews.

#### Data analysis

Thematic analyses are iterative in nature [9] and, to reflect this, transcripts were analysed by two researchers (CS and ML) independently. Initially, broad themes were generated to identify salient constructs. Following this, a consensus meeting was held in which researchers discussed their initial analyses. Any discrepancies between researchers were resolved through discussion. Once agreement was reached, both researchers’ broad themes were synthesised into a table and refined into codes. The primary author and an independent researcher (CS and AS) then reviewed codes and disagreements were discussed until a consensus was reached. To limit researcher bias, data was independently reviewed and analytical notes of researchers own inferences were recorded. In this way, potential biases are documented and can be explored if necessary [10].

### Stage 3: expert feedback and revisions

Draft outcome measures were then submitted to a panel of experts, who were asked to provide feedback and pay particular attention to item clarity, difficulty and relevance. Experts were also asked to delete items they felt inappropriate and add any additional items to ensure the domain to be measured was captured in its entirety.

### Stage 4: pilot study

#### Design

A cross-sectional design was employed in which participants completed two newly developed outcome measures, alongside three other outcome measures. Participants could complete the study in a number of ways depending on their preference. Firstly, in a self-report fashion, without supervision. Secondly, in a self-report fashion, supported by a member of the research team. Finally, in an interview led manner.

#### Participants

Participants were recruited primarily through existing CST groups within a London NHS Foundation Trust and via private and voluntary organisations (e.g. CogsClubs). Four CST groups were contacted and, of these four, two took part in the current study. Ethical approval was granted and the study was adopted for portfolio. The facilitators of these groups (e.g. facilitators of CST groups) identified participants who they deemed eligible to meet the inclusion criteria:

- Diagnosis of dementia according to DSM-IV criteria (American Psychiatric Association, 2000).
- Capacity to give informed consent.

#### Procedure

The researcher initially approached organisations detailing the study to determine whether there might be interest in participation. If group facilitators felt there might be interest, a date to attend the group was arranged. In cases where this was not possible, the study was introduced to the participants by facilitators after they had received all information regarding the study.

Participants were provided with an information sheet and a consent form prior to data collection and informal capacity assessments were conducted with all participants. Four people who were approached were deemed to lack capacity and therefore were not included within the current research. The majority of participants elected to have the researcher present (*n* = 26), hence, arrangements were made to visit participants at convenient dates and times. One CST group decided to complete the study during a break in a CST session (*n* = 4). For this group, the researcher was present to assist, if needed, as they completed the questionnaires. Three participants elected to take the questionnaire pack home with them and return it via post. These participants were provided with a pre-stamped envelope and the work mobile phone number of a member of the research team, should they encounter any difficulty in returning or completing the questionnaires.

#### Analysis

Initially, the homogeneity of domains was tested for both measures using an internal consistency analysis [11]. Following this, convergent validity was assessed using a Pearson’s correlation. Measures chosen to assess convergent validity were:

- Quality of Life in Alzheimer’s Disease Scale (QoL-AD) [12]
- Geriatric Depression Scale SF (GDS) [13].

The QoL-AD was selected as some people able to complete it satisfactorily with a MMSE score of three [14], it has established psychometric properties, is brief in nature and can be completed either in an interview or self-reported format. The GDS has been validated for people with dementia [15] and it is generally accepted that the GDS is an adequate tool to detect depression in people with mild to moderate dementia [16]. Due to the theoretical relationship between quality of life and positive psychology principles, it was hypothesised that a positive correlation would be observed between the PPOM, EID-Q and the QoL-AD. Positive psychology concepts may offer a protective effect against depression and, therefore, it was hypothesised that a negative correlation would be observed between the PPOM, EID-Q and the GDS.

## Results

### Stage 1: literature review

213 results were identified in which a number of positive concepts were explored. However, the majority of these papers were within the caregiving literature. Examples of qualitative studies reporting on people with dementia included a sense of coherence [17], spirituality [18] and hope [19]. A substantial review [20] of the qualitiative literature indicated that resilience was particularly prominent within the qualitative literature, as did a qualitative study on the role of hope. Whilst independence and social engagement were discussed more sparingly and in the context of support or maintaining engagement with previously valued occupations, they may hold important implications for people with dementia. These concepts are discussed in more detail below.

#### Sense of independence and social engagement

The notion of a ‘sense of independence’ rarely appeared in the research literature. However, this may be particularly apt for people with dementia as they often face varying levels of independence within domains such as self-care, mobility and decision making and this can be influenced by how others treat them. More recently, retention of independence has been investigated as a valuable outcome for the maintenance of wellbeing for people with dementia. It can decrease the potential stress felt by carers and delay nursing home entry [21]. However, independence is often defined as a physical ability, for example, the time taken to walk from one place to another. This may be a simplistic view, which does not take into account more subjective views on what it means to be truly independent.

Independence may be closely related to social concepts including engaging with those around you and reciprocity. For example, people with dementia often require assistance from their carer or immediate family to maintain a level of independence they are comfortable with. As dementia progresses, a carer often assumes responsibility for tasks such as activities of daily living [22], financial decisions and advanced planning [23]. This relationship is of paramount importance with a supportive relationship being linked to slower cognitive decline for the person with dementia [24]. It is, therefore, reasonable to suggest that independence for people with dementia may be very closely related and dependent on this relationship. No suitable measures were identified that examined a sense of independence and social engagement, and this may be because previously independence was purely examined as a functional capability. Due to the relationship between these concepts, the decision was made to develop one outcome measure and items were based on discussions in the focus group study.

#### Reslience

Resilience is often used to describe those who display emotional stamina in adverse situations. Its definition is sometimes ambiguous and therefore, the definition used in the current study will be the flexibility in response to changing situational demands [25]. It is noted that whilst some definitions refer explicitly to an adverse event preceding the development of resilience, the definition presented refers to changing situational demands as the precedent for resilience rather than an overtly negative event. Whilst it can be suggested that resilience is not an entirely positive construct, it is included in the current research due to its presence in a recent systematic review of living positively with dementia [26]. More specifically, this review of qualitative literature identified themes of engaging with dementia and ‘facing it and fighting it’ as a form of active perseverance through resilience. An earlier systematic review [26] identified four measures of resilience of varying quality. Of these, the Connor-Davidson Resilience Scale (CD-RISC) [27] was selected for adaption due to its thorough assessment of sensitivity to change and interpretability. However, the CD-RISC is a 25-item measure and, it was decided that this could be too time intensive. As such, the short form version (CD-RISD-SF) was examined and found to have adequate psychometric properties [28].

#### Hope

A substantial qualitative study examined the presence and conceptualisation of hope for people with dementia, for which their findings provide a strong rationale for the applicability of multidimensional hope, rather than goal directed hope [19]. For most, hope was described as an active process whereby a developmental history of ‘learned hope’ remains well preserved and this facilitated a process of ‘keeping going’, viewing difficulties encountered as challenges to overcome. Furthermore, there appeared to be an active re-appraisal of balancing hope and realism. This seemed to indicate that for people with dementia, outcomes hoped for are grounded in realism and generalised in nature, as illustrated by themes of ‘keep living and living well’ and this is consistent with studies of older adults without dementia [29]. Six outcome measures of hope were identified for possible adaption for people with dementia. These scales were then subjected to the Terwee Criteria; a tool for assessing the development procedure of outcome measures [30]. Of the six scales, two emerged as the most rigorously developed: The Hope and Coping in Recovery Measure [31] and the Herth Hope Index. [32] However, as the explicit focus was on hope, the Herth Hope Index was selected as the most appropriate for people with dementia.

### Stage 2: focus group

As no measures of sense of independence and related social concepts were identified within stage 1, specific attention was paid to these concepts during focus groups and interviews. The total sample was 17, in which two focus groups for people with dementia were used (Group 1 *n* = 3; Group 2 *n* = 6) and individual interviews were employed for carers (Table 1).

**Table 1:** Participant demographics

	Age M (SD)	Female *n* (%)	Ethnicity:	Caring for:
PwD^a^ (*n* = 9)	80.56 (±7.13)	7 (77.8%)	Asian: 1 White: 8	n/a
Carer (*n* = 8)	n/a	0:8	Asian: 1 White: 7	Parent: 3 Spouse: 4 Grandparent: 1
TOTAL (*n* = 17)				

^a^Person with Dementia (PwD)

Four overarching themes emerged as central to independence in dementia: 1) independence and interdependence, 2) functional independence, 3) remaining active and 4) social engagement. The first higher order theme illustrates ambiguity in definitions of independence in dementia and indicates that a period of interdependence between a carer and people with dementia can be beneficial for both. The second and third higher order themes reflect the differing domains within the construct of ‘independence’ and suggest that physical and mental ability may have important implications on the retention of independence. This was often compensated for with highly individualistic support provided by carers and a constant reappraisal of abilities and tasks as people with dementia’s ability to engage in such activities declined. The final higher order theme describes the retained desire of people with dementia to engage in social interaction with those around them and illustrates the barriers and facilitators to maintaining this engagement and its relatedness to sense of independence. These themes are discussed briefly below with selected quotes to support their applicability.

#### Independence and interdependence: definitions outside of and within dementia

Participants were initially asked to explore definitions of independence and this was often discussed by carers in the context of isolation from others: ‘I think it’s being able to do things by yourself maybe without the assistance of others or just being free to choose’ (C1). However, when asked to explore what constituted independence for people with dementia, definitions became more varied. Most prominently, participants felt that independence changed as ability to complete activities of daily living declined. This led to interdependence between a person with dementia and a carer. Examples of what this constituted appeared to be the giving of slight assistance to people with dementia so that they could maintain a level of independence that suited their individual ability: ‘Dressing and an occasional prompt… when the automatic is no longer automatic’ (C4).

#### Functional independence: activities of daily living, self-care and decision-making

Independence was discussed within the context of giving the ‘right’ level of support for a person with dementia. Too much could be frustrating for the person with dementia and too little could lead to the person with dementia feeling neglected. Carers often felt that, by adjusting the level of support given to a person with dementia based on their ability, it allowed people with dementia to focus on other decisions: ‘yeah just having sort of basic needs assisted with and then you’ve got extra energy to focus on some other things that might be a bit more challenging’ (C8). The potential for conflict occurred when the person with dementia felt that aspects of self-care were being taken out of their control but still appeared desirous to make a decision even if this was, as seen by the carer, the wrong decision: ‘If I say I don’t think you should… wear that… It’s not suitable for the weather. No he wants to wear it… if I don’t let go he’s very grumpy, upset’ (C2). People with dementia also displayed a retained desire and satisfaction in decision-making*:* ‘because I like to make decisions for myself…’ (P1)

#### Remaining active

Physical ability was implicitly related to maintaining a level of independence: ‘because I don't go out… my legs. I like to go out and get on the bus to Romford… I couldn't walk very far that’s the trouble with my legs’ (P5). As physical ability decreased, complex hobbies were often abandoned ‘I got plenty *(of hobbies)* but I can’t do them now’ (P3) and this could impact upon their sense of independence. An example of this came from a participant who discussed being no longer able to fish: ‘I’ve got to roll myself over to a spot so that I can just get up and not join the fish… it’s impossible’ (P8), suggesting a close relationship between physical abilities and remaining active, and its impact on ability to remain independent. For carers, the need for people with dementia to engage in activities was seen as a form of mental stimulation, to delay dependence for as long as possible: ‘It must keep the bits of her brain that are still functioning more active than they are currently and that just cannot be a bad thing’ (C3).

#### Social engagement

Social concepts were often discussed in close relation with independence. For example: ‘Being able to make decisions yourself without bothering anyone else but at the same time taking their problems into concern’ (P1) and independent social engagement allowed people to feel they had accomplished much: ‘we often say oh I’ll pop out for an hour or so tonight…you feel fulfilled then at the end of the day and you don't need much if you're not talking about anything much’ (P2). It was noted that people with dementia wanted varying levels of engagement and independently choosing their own level of engagement appeared to be the most beneficial: ‘He was quite happy in a large pub where he could wander around and you know just chose who he would talk’ (C1).

Reciprocity was seen by both people with dementia and carers as a means of being useful to a carer in ways that were manageable and was always discussed as an exchange of either activities or sentiment: ‘Being kind to one another’ (P1). A desire to give back was also explored by carers who were aware of the person with dementia’s desire to be reciprocal with them: ‘He knows that it would please me and it would make me happier if he helped. This is why he does it. I wouldn’t say it’s his hobby’ (C2). However, when a person with dementia felt they could not be reciprocal, it could result in feelings of guilt and frustration: ‘I mean bless his heart he says to me now I’ve ruined your life… he gets cheesed off at the fact his daughter is doing what I’m doing’ (C5). This latter example was discussed within the context of a carer doing home improvement tasks around the house; something the person with dementia had been responsible for prior to the onset of dementia. This loss in autonomy resulted in friction within the relationship between the carer and person with dementia.

#### Resilience and hope

Saturation for resilience and hope data was reached very quickly. Resilience appeared to be present for people with dementia and was defined as: ‘Take it as it comes unless you really clever and want to do something but I'm not’ (P2). This attitude of taking life as it comes was discussed with regard to various hospital appointments and health problems. As such, health related resilience might hold more importance than other aspects of resilience for this population and people with dementia noted the additional implications of ageing on health: ‘The older you get the more difficult it becomes’ (P1). In line with previous work, people with dementia viewed hope generally rather than situation specific and as being present on a day-to-day basis: ‘Well I say I hope I get this and I hope I get that’ (P1). The applicability of future oriented hope was also examined by one participant: ‘I feel scared about my future but maybe I don't know if it's further down but just on a day-to-day you are… just worried about the day’ (C2). This may be particularly relevant for people with dementia, as participants with dementia did not discuss future oriented hope.

#### The EID-Q and PPOM

Using the results of stage 1 and 2, 28-items were generated for the Engagement and Independence in Dementia Questionnaire (EID-Q). Due to the inter-relatedness of of independence and social engagement, the decision was made to combine these concepts within one scale. As hope and resilience are more representative of positive psychology traditionally, The Herth Hope Index and CD-RISC-SF were combined and item wording was adapted to create the Positive Psychology Outcome Measure (PPOM).

### Stage 3: expert feedback and revisions

Five experts from the Promoting Independence in Dementia (PRIDE) research programme responded to a request for feedback and revisions. Professions consisted of a professor of old age psychiatry, a reader in clinical psychology, a professor of community care research, a psychologist and a researcher. Results from all responders were pooled into one document using track changes and discussed within the supervisory team, until a consensus as to which suggestions to include as amendments was met. Most suggestions were acted upon to ensure an adequate level of content validity.

#### EID-Q

Responders noted three items that should be removed, as they didn’t appear closely related to the concept in question. Examples of items removed included ‘I feel connected to society’. It was suggested that other items were in need of rewording as they may be too difficult to understand. Six items were reworded to improve clarity. Examples included ‘I can adapt my wishes to be in line with what I can do’, which was replaced by ‘I can make changes to my life to match my ability’ and ‘I can participate in a meaningful conversation’, which was amended to ‘I take part in conversations in ways that I enjoy’. No additional items were suggested but one responder did comment that the overall length might be excessive.

#### PPOM

Responders did not identify any redundant items within the PPOM but suggested that a number of items needed rewording to make them more appropriate for people with dementia. 9 items were reworded and examples included rewording ‘I can see possibilities in the midst of difficulties’ to ‘I can see positive things in difficult situations’ and ‘I am able to adapt’ to ‘I am able to adapt to things’. Other items were suggested as in need of clarifying. For example, ‘I am a strong person’ was amended to ‘I am an emotionally strong person’.

### Stage 4 pilot testing

Thirty-eight people with dementia were approached to complete the novel measures and convergent validity measures. Of these, four lacked the capacity to consent and one declined to take part. This left a sample size of 33 (Table 2). All participants had been diagnosed with dementia and were deemed capable of giving informed consent. Eight participants elected to complete the measures in an interview led manner, whilst 25 elected to complete it in a self-report manner. All data was inputted into an SPSS file on a password-protected computer. Reverse items were recoded on the PPOM and EID-Q so that higher scores indicated a higher level of the domains assessed.

**Table 2:** Participant characteristics

	Total
Gender *n* (*%*)	
Female	19 (57.6)
Male	14 (42.4)
Age *M* (*SD*)	80.18 (8.27)
Marital status *n* (*%*)	
Married	14 (42.4)
Widowed	17 (51.5)
Divorced	2 (6.1)
Residing *n* (*%*)	
Community	30 (90.9)
Residential Facility	3 (9.2)
Ethnicity *n* (*%*)	
White (British)	28 (84.8)
White (other)	2 (6.1)
Black	1 (3)
Asian	2 (6.1)
Dementia diagnosis *n* (*%*)	
Alzheimer’s Disease	13 (39.4)
Vascular Dementia	5 (15.2)
Dementia of mixed etiology	7 (21.2)
Unknown sub-type	8 (24.2)
Time since diagnosis *n* (*%*)	
< 1 year	8 (24.2)
1– 2 years	3 (9.1)
2– 3 years	8 (24.2)
> 3 years	6 (18.2)
Unknown	8 (24.2)
Cholinesterase inhibitors *n* (*%*)	
None	17 (51.5)
Donepezil	4 (12.1)
Memantine	6 (18.2)
Rivastigmine	3 (9.1)
Galantamine	2 (6.1)
Donepezil and Memantine	1 (3)
Other psychotropic medication *n* (*%*)	
None	27 (81.8)
Anti-depressant	6 (18.2)

#### Internal consistency

##### *PPOM*

The first internal consistency analysis revealed that the PPOM had an overall Cronbach alpha level of α = .793, and the subscales were α = .859 for resilience and α = .557 for hope. This highlighted that one or more of the items on the hope subscale may not be pertinent for people with dementia. The resilience subscales’ internal consistency was almost identical to the original, reported as α = 0.85 (Campbell-Sills & Stein, 2007) [24]. Following further analysis, it emerged that the removal of three items on the hope (‘I feel all alone’; ‘I have faith in the future’; ‘I feel scared about the future’;) subscale and two items on the resilience subscale (‘I can achieve my goals’; ‘I am not easily discouraged’) would improve the internal consistency of the PPOM. After removing these items, the overall internal consistency improved to α = .849, with the subscales improving to α = .755 for hope and α = .871 for resilience. This therefore resulted in a 16 item scale, consisting of a hope subscale (8 item) and resilience subscale (8 item).

##### *EID-Q*

Initially the EID-Q had an adequate level of internal consistency (α = .896) and the subscales were also of sufficient value (α = .849 for independence and α = .771 for engagement). However, by removing one item from each subscale (‘there are things I would like to do but can’t; I have good relationships/friendships with others’) internal consistency was raised to α = .868 for independence, α = .775 for social engagement and α = .907 for the scale overall. This resulted in a 26 item scale, consisting of an independence subscale (13 item) and engagement subscale (13 item).

#### Convergent validity

##### *PPOM*

Preliminary indications of convergent validity were found between the PPOM and the GDS. A two-tailed Pearson’s R correlation found a significant, negative correlation between the PPOM and the GDS (*r* = -0.562, *p* = .002) and the hope subscale and the GDS (*r* = -.557, *p* = .001). However, the resilience subscale did not significantly correlate with GDS (r = -.312, *p* = .088). An additional Pearson’s R correlation was performed to assess the relationship between scores on the QoL-AD and the PPOM but no significant correlations were found.

##### *EID-Q*

As with the PPOM, a two-tailed Pearson’s R correlation was performed for both subscales and the EID-Q scale and the GDS. Significant correlations were found between the independence subscale (*r* = -.447, *p* = .012) and GDS, the engagement subscale (*r* = -.430, *p* = .016) and the GDS and the EID-Q and the GDS (*r* = -.461, *p* = .009). This again indicates a negative correlation between the sense of independence, social engagement and depression. Pearson’s R correlations between the EID-Q and the QoL-AD indicated an emerging positive relationship between independence, engagement and quality of life. Firstly, the total QoL-AD score was found to be positively correlated with the independence subscale (*r* = .497, *p* = .005), engagement was correlated with the QoL-AD (*r* = .586, *p* = .001) and the EID-Q was found to be positively correlated with the total QoL-AD score (*r* = .557, *p* = .001) (Table 3).

**Table 3:** Pearson’s R correlations between measures

	GDS	QoL-AD
PPOM Total:	r = -.562, *p* = .02	*-*
PPOM: Hope	r = -.557, *p* = .001	-
PPOM: Resilience	r = -.312, *p* = .088	-
EID-Q Total	r = -.461, *p* = .009	r = .557, *p* = .001
EID-Q: Independence	r = -.447, *p* = .012	r = .497, *p* = .005
EID-Q: Engagement	r = -.430, *p* = .016	r = .586, *p* = .001

## Discussion

### Summary of results

This is, to our knowledge, the first study to report on the development of positive psychology outcome measures for people with dementia. The Positive Psychology Outcome Measure (PPOM) and the Engagement and Independence in Dementia Questionnaire (EID-Q) were developed using a robust four-stage procedure resulting in robust positive psychology outcome measures (Additional file 1). Potentially relevant concepts and related outcome measures were sourced and examined in the current literature. Content validity was provided during focus group studies and expert revision. Finally, a small-scale pilot established adequate psychometric data for the new measures. The two new measures were easy to use and acceptable to participants, with most being able to give an informed opinion as to the use of these concepts in everyday life.

### Findings in the context of the literature

Increasingly, the aim of psychosocial research for people with dementia is to maintain independence through interventions including exercise [33] and occupational therapy [34]. However, within these studies independence is defined in varying forms and usually as a functional ability, for example, the time taken to walk from one place to another or the ability to dress, both without the assistance from others. Whilst there appears to be an implicit expectation that improving independence will increase wellbeing, the observed relationship can be ambiguous [35]. The current study used definitions of independence as discussed by people with dementia in a qualitative setting and as such, the EID-Q is a more holistic measure of a sense of independence, rather than a measure of an operant capability.

The importance of the relationship between a person with dementia and their carer has previously been explored with a reciprocal relationship being highlighted as an important feature [36] and it has been proposed that reciprocity is a potential means of mitigating a loss of autonomy [37]. This reciprocal relationship refers to the ability of the person with dementia to provide assistance to their carer in ways that they feel are beneficial to them, in exchange for the carer providing means of assistance to them. Reciprocal relationships have been found to have numerous health benefits including reducing risk of stroke [38] and increasing psychological wellbeing for older adults [39].

Accounts of hope within the current study were consistent with previous findings in which future oriented hope appeared to be less appropriate for people with dementia [40] and accounts of resilience appeared to support the definition of adapting to changing situational and personal demands. Evidence for the relationship between hope and depression was also documented here and is consistent with the theory that positive psychology concepts may protect against depression [41, 42]. However no significant correlation was found between the PPOM and QoL-AD, possibly indicating hope and resilience may be distinct from quality of life.

Resilience as a positive psychology trait is not without issue. It is generally accepted that resilience is not a construct entirely characteristic of positive psychology but it was included within the current research due to its presence in the largest qualitative study of positive psychology and people with dementia to date [20]. Furthermore, positive psychology seeks to provide a balance and to exclude such a salient characteristic could leave to a tyranny of positives in which potentially beneficial concepts for wellbeing are not examined, because it is not seen as a completely positive trait.

Currently, there is a wealth of positive psychology outcome measures in use for other populations [43] but this approach is only just beginning to be applied to those with dementia research. There is an increasing recognition that positive factors can act as a protective agent in the development of negative emotional states including depression, and this has been exampled in the dementia caregiving literature [44], where positive aspects of caregiving has been proposed as a buffer the negative consequences of caregiving such as depression, burnout and medical risk. The positive psychology approach to dementia reflects an asset-based standpoint, in which the retained capabilities and strengths that people with dementia exhibit are examined. These retained capabilities or strengths may be an important contributing factor to a person’s wellbeing and interventions could be designed to bolster or maintain these strengths over time.

### Methodological problems and limitations

Preliminary psychometric properties were established with a relatively small sample size, increasing the risk of a type II error. Also, the format in which measures were delivered may have impacted upon the instances of missing data. 25 participants elected to complete the measures in a self-report manner and these participants were more likely to miss items than those who completed the study in an interview led manner. This may have been a formatting issue or that participants felt unable to make judgments on particular items without guidance. Certainly within the interview led sample, participants struggled to answer future oriented questions within the hope subscale for the PPOM. This is supported by previous research in which goal oriented hope was found to be lacking for people with dementia [19]. Future oriented questions regarding hope were subsequently removed from the measure. Furthermore, it was noted that carers often had differing perspectives to people with dementia. This was particularly evident for the item ‘I can look after myself as much as I need to’ and it is unclear how much this affected the self-report sample.

We did not collect information with regard to cognition as the qualitative literature suggests that people with dementia experience these positive emotions throughout the course of dementia [20] and as such scores on a cognition test would not be relevant to these measures. However, all participants here had capacity to provide informed consent and so it can be assumed they were in the more mild stages of dementia. We therefore cannot make assumptions about the experience of positive emotions in later stages of dementia. This is something future researchers may wish to examine further.

Finally, it must be noted that a proportion of our participants were also receiving interventions including Cognitive Stimulation Therapy. These participants may differ slightly to those not engaging in activities but this is mitigated by the pilot study sample, for which participants who were not receiving an intervention were also involved. This limitation will be addressed in a further larger scale study.

### Future research

As this study only provided tentative psychometric properties for the PPOM and EID-Q, the next stage is a larger study and further psychometric analysis, which is currently ongoing, and will allow the testing of the measures test-retest reliability, discriminant validity, factor structure and responsiveness, This will ensure that the developed measures can be applied within dementia services and research across the country.

Positive psychology is a broad discipline and there appears to be a need to formulate a theoretical model of this approach tailored specifically for dementia, to recognise the unique difficulties and capabilities this population has. This has, to some extent, been established within the caregiving literature with a focus on positive experiences of caregiving [45, 46] but is currently lacking within the dementia literature.

The evaluation of positive psychology concepts share difficulties with the appraisal of quality of life as it is argued that, below a certain level of function, people with dementia struggle to accurately appraise this [47]. Nevertheless, this study illustrates that, whilst positive psychology concepts require complicated appraisals, people with dementia appear able to give a view to this. As such, future quantitative research is needed to examine other concepts that may hold significance for people with dementia including love and humour.

## Conclusion

Two positive psychology measures were developed using a robust, iterative process. The EID-Q and PPOM demonstrated acceptable internal consistency and convergent validity. However these psychometric properties were established using a small sample. Further psychometric analysis is needed before such measures can be used in psychosocial research to improve our knowledge and understanding of wellbeing for people with dementia.

## Additional file

Additional file 1: The Engagement and Independence in Dementia Questionnaire (EIDQ) and Positive Psychology Outcome Measure (PPOM). Description of Data: The EID-Q (26-item) and the PPOM (16-item). Both are answered on a 5-point Likert scale, with a timescale of the previous month. (DOCX 107 kb)

## References

[1] Shankar KK, Walker M, Frost D, Orrell MW (1999). The development of a valid and reliable scale for rating anxiety in dementia (RAID). Aging Ment Health.

[2] Alexopoulos GS, Abrams RC, Young RC, Shamoian CA (1988). Cornell scale for depression in dementia. Biol Psychiatry.

[3] Cummings JL (1997). The neuropsychiatric inventory: assessing psychopathology in dementia patients. Neurology.

[4] Hurt C, Bhattacharyya S, Burns A, Camus V, Liperoti R, Mariott A (2008). Patient and caregiver perspectives of quality of life in dementia: an investigation of the relationship to behavioural and psychological symptoms in dementia. Dement Geriatr Cogn Disord.

[5] Seligman ME, Steen TA, Park N, Peterson C (2005). Positive psychology progress: empirical validation of interventions. Am Psychol.

[6] Clarke C, Wolverson E, Stoner C, Spector A, Wolverson E, Clarke C (2016). Overview and ways forward for a positive psychology approach to dementia. Positive psychology approaches to dementia.

[7] Seligman ME (1998). The President's address. American psychological association.

[8] Padgett DK (1998). Qualitative methods in social work research: challenges and rewards.

[9] Braun V, Clarke V (2006). Using thematic analysis in psychology. Qual Res Psychol.

[10] Saldana J (2009). The coding manual for qualitative researchers.

[11] Cronbach LJ (1951). Coefficient alpha and the internal structure of tests. Psychometrika.

[12] Logsdon RG, Gibbons LE, McCurry SM, Teri L (2002). Assessing quality of life in older adults with cognitive impairement. Psychosom Med.

[13] Sheikh JI, Yesavage JA (1986). Geriatric depression scale (GDS): recent evidence and development of a shorter version. Clin Gerontol.

[14] Thorgrimsen L, Selwood A, Spector A, Royan L, de Madariaga LM, Woods RT (2003). Whose quality of life is it anyway? the validity and reliability of the quality of life-Alzheimer's disease (QoL-AD) scale. Alzheimer Dis Assoc Disord.

[15] Lesher EL, Berryhill JS (1994). Validation of the geriatric depression scale: short form among inpatients. J Clin Psychol.

[16] Feher EP, Larrabee GJ, Crook TH (1992). Factors attentuating the validity of the geriatric depression scale in a dementia population. J Am Geriatr Soc.

[17] Lillekroken D, Hauge S, Slettebo A (2015). Enabling resources in people with dementia: a qualitative study about nurses’ strategies that may support a sense of coherence in people with dementia. J Clin Nurs.

[18] Dalby P, Sperlinger D, Boddingston S (2011). The lived experience of spirituality and dementia in older people living with mild to moderate dementia. Dementia.

[19] Wolverson EL, Clarke C, Moniz-Cook E (2010). Remaining hopeful in early-stage dementia: a qualitative study. Aging Ment Health.

[20] Wolverson EL, Clarke C, Moniz-Cook ED (2015). Living positively with dementia: a systematic review and synthesis of the qualitative literature. Aging Ment Health.

[21] Spillman BC, Long SK (2009). Does high caregiver stress predict nursing home entry?. Inquiry.

[22] Holst G, Edberg A (2011). Wellbeing among people with dementia and their next of kin over a period of three years. Scan J Caring Sci.

[23] Livingston G, Leavey G, Manela M, Livingston D, Rait G, Sampson E (2010). Making decisions for people with dementia who lack capacity: qualitative study of family carers in UK. BMJ.

[24] Norton MC, Piercy KW, Rabins PV, Green RC, Breitner JC, Østbye T (2009). Caregiver-recipient closeness and symptom progession in Alzheimer disease. The cache county dementia progession study. J Gerontol B Psychol Scie Soc Sci.

[25] Tugade MM, Fredrickson BL, Feldman BL (2004). Psychological resilience and positive emotional granularity: examining the benefits of positive emotions on coping and health. J Personal.

[26] Stoner CR, Orrell M, Spector A (2015). Review of positive psychology outcome measures for chronic illness, traumatic brain injury and older adults: adaptability in dementia?. Dement Geriatr Cogn Disord.

[27] Connor KM, Davidson JR (2003). Development of a new resilience scale: the connor-davidson resilience scale (CD-RISC). Depress Anxiety.

[28] Campbell-Sills L, Stein MB (2007). Psyhometric analysis and refinement of the Connor-Davidson resilience scale (CD-RISC): validation of a 10-item measure of resilience. J Trauma Stress.

[29] Herth K (1993). Hope in older adults in community and institutional settings. Issues Ment Health Nurs.

[30] Terwee CB, Bot SM, de Boer MR, van der Windt DM, Knol DL, Dekker J (2007). Quality criteria were proposed for measurement properties of health status questionnaires. J Clin Epidemiol.

[31] Bradshaw SD (2014). The development of the hope and coping in recovery measure (HCRM). J Groups Addict Recover.

[32] Herth K (1992). Abbreviated instrument to measure hope: development and psychometric evaluation. J Adv Nurs.

[33] Hogervorst E, Clifford A, Stock J, Xin X, Bandelow S (2012). Exercise to prevent cognitive decline and Alzheimer's disease: for whom, when, what and (most importantly) how much?. J Alzheimers Dis Parkinsonism.

[34] Gitlin LN, Winter L, Dennis MP, Hodgson N, Hauck WW (2010). Biobehavioural home-based intervention and the wellveing of patients with dementia and their caregivers: the COPE randomised trial. JAMA.

[35] Baines S, Saxby P, Ehlert K (1987). Reality orientation and reminiscence therapy: a controlled cross-over study of elderly confused people. Br J Psychiatry.

[36] Førsund LH, Skovdahl K, Kiik R, Ytrehus S (2015). The loss of a shared lifetime: a qualitative study exploring spouses' experiences of losing couplehood with their partner with dementia living in institutional care. J Clin Nurs.

[37] Vernooij-Dassen M, Leatherman S, OldeRikkert M (2011). The fragile balance between receiving and giving. BMJ.

[38] Boden-Albala B, Litwak E, Elkind MS, Rundek T, Sacco RL (2005). Social isolation and outcomes post-stroke. Neurology.

[39] Park NS (2009). The relationship of social engagement to psychological well-being of older adults in assisted living facilities. J Appl Gerontol.

[40] Wolverson E, Clarke C, Wolverson E, Clarke C (2016). Hope and dementia. Positive psychology approaches to dementia.

[41] Edward K (2016). Resilience: a protector from depression. J Am Psychiatr Nurses Assoc.

[42] Cheavens J, Snyder CR (2000). Hope and depression: light through the shadows. Handbook of hope.

[43] Daaleman TP, Frey BB, Wallace D, Studenski SA (2002). The spirituality index of well-being: development and testing of a new measure. J Fam Pract.

[44] Tremont G (2011). Family caregiving in dementia. Med Health.

[45] Zarit SH (2012). Positive aspects of caregiving: more than looking on the bright side. Aging Ment Health.

[46] Carbonneau H, Caron C, Desrosiers J (2010). Development of a conceptual framework of positive aspects of caregiving in dementia. Dementia.

[47] Moyle W, Gracia N, Murlfield JE, Griffiths SG, Venturato L (2011). Assessing quality of life in older people with dementia in long-term care: a comparison of two self-report measures. J Clin Nurs Title.

