# Supplementary material for: The development and preliminary psychometric properties of two positive psychology outcome measures for people with dementia: the PPOM and the EID-Q
Source: BMC Geriatr. 2017 Mar 21;17:72. doi: 10.1186/s12877-017-0468-6 (PMC5361794; doi:10.1186/s12877-017-0468-6)
Supplement: Additional file 1: — The Engagement and Independence in Dementia Questionnaire (EIDQ) and Positive Psychology Outcome Measure (PPOM). Description of Data: The EID-Q (26-item) and the PPOM (16-item). Both are answered on a 5-point Likert scale, with a timescale of the previous month. (DOCX 107 kb) [file 12877_2017_468_MOESM1_ESM.docx]

| **Additional File 1**  ***The Engagement and Independence in Dementia Questionnaire (EID-Q) and Positive Psychology Outcome Measure (PPOM)***  **Instructions**  We would like to know how you have been feeling over the **past month**. Please answer the below questions by **circling one number** (0, 1, 2, 3 or 4) that most closely reflects how you have felt for each question. Please answer all the questions. If you are unsure, circle the number that is your best guess. | | | | | |
| --- | --- | --- | --- | --- | --- |
| **EID-Q** | | | | | |
|  | **Not true at all** | **Rarely true** | **Sometimes true** | **Often true** | **True nearly all the time** |
| 1. I can look after myself as much as I need to | 0 | 1 | 2 | 3 | 4 |
| 2. I have people who I can talk to if I need to | 0 | 1 | 2 | 3 | 4 |
| 3. I have hobbies/ activities that I enjoy doing | 0 | 1 | 2 | 3 | 4 |
| 4. I have a role in my social circle | 0 | 1 | 2 | 3 | 4 |
| 5. I am a burden to others | 0 | 1 | 2 | 3 | 4 |
| 6. I enjoy conversations with others | 0 | 1 | 2 | 3 | 4 |
| 7. I can make my own decisions as much as I’d like to | 0 | 1 | 2 | 3 | 4 |
| 8. There are people I could ask for help if I need to | 0 | 1 | 2 | 3 | 4 |
| 9. I’m confident in making decisions | 0 | 1 | 2 | 3 | 4 |
| 10. I am often ignored by those around me | 0 | 1 | 2 | 3 | 4 |
| 11. I can do activities that are important to me | 0 | 1 | 2 | 3 | 4 |
| 12. I can get in touch with friends/ family if I need to | 0 | 1 | 2 | 3 | 4 |
| 13. People take decisions away from me | 0 | 1 | 2 | 3 | 4 |
| 14. My friends/ family care about me | 0 | 1 | 2 | 3 | 4 |
| 15. I can arrange my life in a way that suits me best | 0 | 1 | 2 | 3 | 4 |
| 16. I can help the people I care about | 0 | 1 | 2 | 3 | 4 |
| 17. I feel I am active in everyday life | 0 | 1 | 2 | 3 | 4 |
| 18. I can take part in groups/ activities with others | 0 | 1 | 2 | 3 | 4 |
| 19. I can adapt my wishes to be in line with what I can do | 0 | 1 | 2 | 3 | 4 |
| 20. I feel that my friends/ family want to spend time with me | 0 | 1 | 2 | 3 | 4 |
| 21. I can make changes to my life to match my abilities | 0 | 1 | 2 | 3 | 4 |
| 22. I can confide in my friends/ family | 0 | 1 | 2 | 3 | 4 |
| 23. I can get myself food if I need to | 0 | 1 | 2 | 3 | 4 |
| 24. I can help my friends/ family as much as I would like | 0 | 1 | 2 | 3 | 4 |
| 25. I keep myself busy with activities/ hobbies | 0 | 1 | 2 | 3 | 4 |
| 26. I feel connected to others | 0 | 1 | 2 | 3 | 4 |

| We would like to know how you have been feeling over the **past month**. Please answer the below questions by **circling one number** (0, 1, 2, 3 or 4) that most closely reflects how you have felt for each question. Please answer all the questions. If you are unsure, circle the number that is your best guess. | | | | | |
| --- | --- | --- | --- | --- | --- |
| **PPOM** | | | | | |
|  | **Not true**  **at all** | **Rarely true** | **Sometimes true** | **Often true** | **True nearly all the time** |
| I have a positive outlook on life | 0 | 1 | 2 | 3 | 4 |
| I can see positive things in difficult situations | 0 | 1 | 2 | 3 | 4 |
| I can recall happy/ joyful times | 0 | 1 | 2 | 3 | 4 |
| I have inner strength | 0 | 1 | 2 | 3 | 4 |
| I can give and receive care/ love | 0 | 1 | 2 | 3 | 4 |
| I have a sense of direction in life | 0 | 1 | 2 | 3 | 4 |
| I believe that each day has potential | 0 | 1 | 2 | 3 | 4 |
| My life has value and worth | 0 | 1 | 2 | 3 | 4 |
| I am able to adapt to things | 0 | 1 | 2 | 3 | 4 |
| I am able to deal with whatever happens | 0 | 1 | 2 | 3 | 4 |
| I am able to see the humorous side | 0 | 1 | 2 | 3 | 4 |
| I can cope with stress well | 0 | 1 | 2 | 3 | 4 |
| I can bounce back | 0 | 1 | 2 | 3 | 4 |
| I can stay focused | 0 | 1 | 2 | 3 | 4 |
| I am an emotionally strong person | 0 | 1 | 2 | 3 | 4 |
| I can handle unpleasant feelings | 0 | 1 | 2 | 3 | 4 |
